# Combination of β-mannanase plus multi-carbohydrase complex in simple or complex post-weaned pig diets on nutrient metabolism and gut health

**DOI:** 10.3389/fvets.2024.1404382

**Published:** 2024-08-16

**Authors:** Gabriela M. Galli, Ines Andretta, Camila L. Carvalho, Thais B. Stefanello, Maria S. C. Mendéz, Ricardo E. Mendes, Vitor W. Horn, Marcos Kipper

**Affiliations:** ^1^Department of Animal Science, Universidade Federal do Rio Grande do Sul, Porto Alegre, Brazil; ^2^Athens Veterinary Diagnostic Laboratory, College of Veterinary Medicine, University of Georgia, Athens, GA, United States; ^3^Laboratory of Veterinary Pathology, Instituto Federal Catarinense, Concórdia, Brazil; ^4^Elanco Animal Health, São Paulo, Brazil

**Keywords:** arabinofuranosidases, β-glucanase, β-mannanase, digestibility, diet composition, intestinal health, swine, xylanases

## Abstract

This study was conducted to evaluate whether adding β-mannanase alone or in combination with a multi-carbohydrase complex to simple and complex diets could improve diet digestibility, nutrient and energy metabolism, and gut health in weaned pigs. Thirty pigs (7.9 kg ± 0.851 kg) weaned at 28 days were randomly split into a 2 × 3 factorial arrangement, considering a simple (corn and soybean meal-based diet) or complex diet (13% point reduction in inclusion of soybean meal, 5% of whey power, and 2.5% of spray-dried plasma compared to the simple diet) and diet without any addition (control) or the addition of β-mannanase (BM; 0.300 g/kg of the diet) or β-mannanase plus a multi-carbohydrase complex blend such as xylanase, β-glucanase, and arabinofuranosidases (BM + MCC; 0.300 + 0.050 g/kg of the diet) for 17 days post-weaned. Total fecal and urine samples were collected on days 11–17. Fecal samples were collected from all pigs to identify fecal biomarkers using commercial ELISA tests. Blood samples were collected from all pigs at the end of the experimental period to assess serum concentrations of acute-phase proteins. All pigs were euthanized on day 18 for intestinal tissue collection. The simple diet had greater (*p* < 0.05) protein digestibility and metabolizability coefficients than the complex diet. Greater (*p* < 0.05) energy digestibility and energy metabolizability coefficients were observed in the BM and BM+ MCC compared to the control diet. On average, BM improved by 64 kcal/kg and BM + MCC improved by 100 kcal/kg of metabolizable energy. Furthermore, the addition of BM and BM + MCC to the diets led to lower fecal moisture and fecal output. Moreover, the BM and BM + MCC diets also reduced fecal calprotectin concentrations by 29 and 46%, respectively, compared to control pigs (*p* < 0.001). We conclude that simple diets are a suitable alternative to complex diets, without compromising the nutrient digestibility and gut health of post-weaned pigs. The addition of exogenous enzymes improves nutrient and energy utilization, as well as the absorption area, and decreases calprotectin concentrations.

## 1 Introduction

Weaning is a critical stage in a pigs’ life that causes morphological and functional changes in the intestine where most of the nutrients are being digested and absorbed (1). Furthermore, various other stressors induce post-weaned growth depression, such as separation from the sow and littler, transition from a liquid to a solid diet, and hierarchy formation. The endogenous secretion of several enzymes and hydrochloric acid is considered insufficient during this phase. Furthermore, microbial dysbiosis at weaning is one of the leading causes of post-weaning diarrhea, which results in significant economic losses. Therefore, most post-weaning diarrhea is caused by *Escherichia coli*, enterotoxigenic *E. coli* (ETEC) strains mostly, but also by other pathogens such as enteroadherent strains of *E. coli, Salmonella enterica*, or rotavirus (2).

Pig diets are typically mainly composed of corn and soybean meal. Despite their high nutritional value, these ingredients contain several anti-nutritional factors that can impair animal metabolism, such as fibers found in cell walls (3). Certain anti-nutritional factors can be reduced through the treatment of these ingredients, but not all are effectively managed by the typical processes employed in the industry. These components (arabinoxylans, xylose, phytate, and mannans) are difficult to manage during weaning when pigs face many other challenges to their gut health (4). Simple diets, which are formulated with a higher inclusion of soybean meal, may be particularly challenging, and for that reason, the use of complex diets is considered necessary during this phase (5). However, complex diets are more expensive because of the greater inclusion of highly digestible feed ingredients such as dried animal plasma, whey, and soy isolates. The literature on the use of simple or complex diets in the post-weaned period is limited and controversial, especially for processed soy protein (5, 6).

Among the various commercially available enzymes, three stand out for their complementary activities in energy utilization and their effect on pig gut health in the post-weaned period. β-mannanases are major mannan-degrading enzymes and are considered energy-saving because they reduce the cost of unnecessary immune response activation (7, 8). Mannans are present, especially in soybeans, and can cause intestinal inflammation, called the feed-induced immune response (FIIR) (7). Furthermore, arabinoxylans and xylose are anti-nutritional factors present in all vegetable ingredients, including corn, wheat bran, and soybean meal, which can reduce nutrient digestibility, increase endogenous losses, and increase digestive viscosity (9). In this context, and by different action mechanisms, the inclusion of β-mannanases, arabinofuranosidases, and xylanases in swine diets may improve energy utilization and gut health (9–11).

However, more information on this matter is important to help nutritionists choose between simple and complex diets because different formulations significantly alter the availability of substrates for enzyme activity and, consequently, can affect the potential benefit obtained from enzyme addition. Both enzyme additions are used with an energy matrix in the feed formulation, which leads some nutritionists to frequently ask questions regarding their potential use in combination. As these additives perform different functions, such as saving energy that would otherwise be spent on the immune response (β-mannanases) or releasing nutritional components for absorption (arabinofuranosidases and xylanases), their effects may be additive. Therefore, this study was conducted to evaluate the impact of adding β-mannanase alone or in combination with a multi-carbohydrase complex, such as xylanase, β-glucanase, and arabinofuranosidases, in simple or complex diets on diet digestibility, nutrient and energy metabolism, as well as gut health in post-weaned pigs.

## 2 Materials and methods

### 2.1 Enzymes

Both the enzymes used in this study are commercially available. β-mannanase (Hemicell HT, Elanco Animal Health, São Paulo, Brazil) is an exogenous enzyme derived from *Paenibacillus lentus* fermentation. The multi-carbohydrase complex blend (Rovabio Advance, Adisseo, São Paulo, Brazil) was made up of arabinofuranosidases, β-glucanase, and xylanases produced by *Talaromyces versatilis* fermentation.

### 2.2 Animals, diets, and experimental procedures

The experiment was carried out in the experimental pig house at the Federal University of Rio Grande of Sul, located in Porto Alegre, Rio Grande of Sul, Brazil. Thirty male pigs (7.903 kg ± 0.851 kg; Large white × Landrace) were weaned at 28 days and individually housed in metabolism crates (1.12 meters long x 0.60 meters wide x 1.92 meters high) and split into one of the six treatments with five replicates. The pigs selected for this trial had not previously received antibiotic treatment. The trial lasted for 17 days, which corresponded to 10 days of adaptation and 7 days of sample collection. The temperature, humidity, and air circulation were controlled during the entire period to ensure comfortable environmental conditions for the pigs.

Pigs were randomly assigned to one of six treatments immediately after weaning and distributed in a 2 × 3 factorial arrangement, with two diet types: simple or complex diets and three enzyme additions: control (no addition), β-mannanase (0.300 g/kg of the diet; BM), or β-mannanase plus a multi-carbohydrase complex, such as xylanase, β-glucanase, and arabinofuranosidases (0.300 g/kg + 0.050 g/kg of the diet; BM + MCC).

Pigs were randomly assigned to one of three groups: control (no addition), addition of β-mannanase (0.300 g/kg of the diet; BM), and β-mannanase plus multi-carbohydrase complex, such as xylanase, β-glucanase, and arabinofuranosidases (0.300 g/kg + 0.050 g/kg of the diet; BM + MCC).

The ingredient composition and calculated nutrient content of the diets provided during the post-weaned period are presented in Table 1. The experimental feeds were formulated for the minimum cost solution to meet at least the nutritional requirements recommended by the Brazilian Tables for Poultry and Swine (12). The same reference was used for ingredient composition, except for soybean meal and corn, for which the total energy and crude protein contents were analyzed and later used to estimate metabolizable energy and digestible amino acid levels (12).

**Table 1:** Diet formulation and chemical composition as-fed basis according to the diet type and enzyme addition^1^.

	Experimental treatments					
Diet type	Simple			Complex		
Enzyme addition ^1^	Control	BM	BM + MCC	Control	BM	BM + MCC
Ingredients, %						
Corn	54.29	54.29	54.29	57.08	57.08	57.08
Soybean meal 46%	25.00	25.00	25.00	12.00	12.00	12.00
Soybean oil	2.519	1.435	1.435	1.607	0.522	0.522
Soybean protein isolate 80%	4.303	4.303	4.303	8.130	8.130	8.130
Meat and bone meal	5.000	5.000	5.000	5.000	5.000	5.000
Spray-dried plasma	2.500	2.500	2.500	5.000	5.000	5.000
Whey powder	5.000	5.000	5.000	10.000	10.000	10.000
Salt	0.083	0.083	0.083	–	–	–
Limestone	0.421	0.421	0.421	0.408	0.408	0.408
Premix^2^	0.500	0.500	0.500	0.500	0.500	0.500
L-Lysine	0.171	0.171	0.171	0.110	0.110	0.110
DL-Methionine	0.111	0.111	0.111	0.100	0.100	0.100
L-Threonine	0.088	0.088	0.088	0.053	0.053	0.053
Phytase^3^	0.005	0.005	0.005	0.005	0.005	0.005
Inert (washed sand)	–	1.054	1.049	–	1.054	1.049
β-mananase^4^	–	0.030	0.030	–	0.030	0.030
Multi-enzyme blend^5^	–	–	0.005	–	–	0.005
Calculated composition						
Crude protein, %	24.08			23.80		
Digestible lysine %	1.346			1.346		
Metabolizable. energy, kcal/kg	3,375	3,285	3,285	3,375	3,285	3,285
Total calcium, %	0.973			0.973		
Total phosphorus, %	0.644			0.644		
Available phosphorus, %	0.528			0.528		

1 Enzyme addition: Without any addition (Control) or addition with β-mannanase (BM; 0.300 g/kg of the diet) or β-mannanase plus a multi-carbohydrase complex containing xylanase, β-glucanase, and arabinofuranosidases (BM + MCC; 0.300 + 0.050 g/kg of the diet).

2 Premix with vitamins and minerals per kg/feed. Vitamin A:12.800.000,0 UI/kg; vitamin B1: 1.800,00 mg/kg; vitamin B2:11.40 g/kg; vitamin K3 6.600,00 mg/kg; vitamin B6: 3.600,00 mg/kg; vitamin B12 52.000,00 mcg/kg; vitamin D3:2.650.00,00 UI/kg; vitamin E: 72.200,00 UI/kg; folic acid 600.00 mg/kg; niacin: 80.00 g/kg; biotin 200.00 mg/kg; pantothenic acid: 40.00 g/kg. calcium 90 g/kg; phosphorus 20 g/kg; sodium 35 g/kg; copper 1,000 mg/kg; iron 1,000 mg/kg; iodine 20 mg/kg; managanese 500 mg; zinc 15 g/kg.

3 Natuphos (BASF Corporation, São Paulo, Brazil).

4 Hemicell HT (Elanco Animal Health, São Paulo, Brazil).^5^Rovabio Advance (Adisseo, São Paulo, Brazil).

The simple and complex diets were formulated independently. The formulation procedures differed in the proportion of soybean meal (25 and 12% for simple and complex diets, respectively), whey powder (5 or 10%), and spray-dried plasma (2.5 or 5%) were defined as fixed objectives. The inclusion of other ingredients in the control diet was determined without restrictions by the formulation procedure. Three modifications were performed using the control formula to obtain the enzyme addition treatments. First, β-mannanase or β-mannanase plus a multi-carbohydrase complex were included in the formulas depending on the treatment. In addition, the inclusion of soybean oil in the enzymes’ diets was adjusted to reduce metabolizable energy by 90 kcal/kg, which is comparable to the energy matrix commonly attributed to the enzymes in practical conditions. The entire energy matrix attributed to the enzymes was applied to reduce soybean oil (which has high availability and contributes only to energy in the formula) to avoid the bias of modifying other ingredients that would modify the substrate availability. Later, a final adjustment was performed using an inert material to complete the formula (i.e., washed sand was added until the sum of the ingredient levels reached 100%). Phytase was included (with a matrix for Ca and P of 500 FTU/kg) in all treatments to better represent standard commercial feeding programs. The analyzed composition of feed samples was validated before the trial.

### 2.3 Data collection

#### 2.3.1 Digestibility and metabolism

Metabolism crates were equipped with trays for the total collection of feces and a system for the total collection of urine. Water and mash-form feed were provided *ad libitum* during the adaptation period. During the collection period, the pigs received feed according to metabolic body weight (2.6 × the estimated maintenance requirement) (13). Leftovers (that remained in the feeder at the end of the day or were found in the tray) were collected, weighed, and stored. These samples were analyzed for dry matter, and the results were used to calculate the daily feed intake.

Feed samples were collected daily, identified, and stored in a freezer until further analysis. Feces and urine were collected twice a day (8:00 a.m. and 5:30 p.m.) in trays and collecting systems installed in the crates. The beginning and end of the collection periods were defined using an indigestible marker (0.8% ferric oxide) mixed in the diets. All the samples were stored in plastic containers, identified by the experimental unit, and stored in a freezer.

At the end of the experimental period, the fecal and urine samples were thawed at room temperature, weighed, and homogenized. Samples from each experimental unit were collected and lyophilized. Then, the samples of feed, feces, and urine were analyzed for dry matter (dried in an oven at 105°C), nitrogen (micro Kjeldahl method), and gross energy (calorimetric pump), following the procedures described by AOAC (14). The coefficients of digestibility (dry matter, protein, and energy) and metabolizability (protein and energy), in addition to the apparent metabolizable energy values, were calculated from data obtained according to the equations provided by Sakomura and Rostagno (15). The manure production was also estimated as the total volume of all urine and feces collected during the digestibility period.

#### 2.3.2 Feed retention rate and fecal moisture content

The time spent from the first consumption of feed with ferric oxide and the appearance of marked feces were registered at the beginning and end of each trial to obtain the feed retention rate the procedure was determined following the procedure described by Moore and Winter (16), with minor modifications. The feces collected each day were weighed and homogenized. A sample corresponding to 20% of the total weight was retained to determine the dry matter content (dried in an oven at 105°C for 8 h).

#### 2.3.3 Acute phase proteins

At the end of the experiment, all pigs were fasted for 8 h and blood was collected in the vena cava in vacutainer tubes without anticoagulant. The samples were identified and held in the thermal box with ice for 40 min before centrifugation. Samples were centrifuged at 3500 rpm for 10 min and the serum was separated, collected, and frozen (−20°C) for acute phase proteins analysis. Serum haptoglobin, transferrin, and C-reactive protein concentrations were determined by sodium dodecyl sulfate-polyacrylamide gel electrophoresis (17). The molecular weight and protein fraction concentrations were determined using computer densitometry (Shimadzu 9,301 PC, Shimadzu Corp, Kyoto, Japan) with a basic scanner. Proteins were identified using biomarkers (Sigma Marker, Sigma-Aldrich Biotechnology LP, Germany). For densitometric examination of the protein bands, reference curves were created from the reading of the standard marker. Afterward, the concentrations were normalized to the total protein serum concentration determined using commercial reagents (Wiener lab, São Paulo, Brazil).

#### 2.3.4 Intestinal morphology and rupture resistance

The pigs were slaughtered at the end of the experiment following the animal welfare and euthanasia standards outlined in the CONCEA euthanasia practice guidelines (18) using electrical stunning methods. Intestinal samples (4 cm distal to the stomach for the duodenum, mid jejunum, and 4 cm distal to the jejunum for the ileum) were collected and stored in 10% formaldehyde solution. Histological slides were prepared and stained with hematoxylin and eosin (H&E). The height and perimeter of the villi and the depth of the intestinal crypts were determined in the intestinal fragments using the methodology described by Caruso and Demonte (19). Histological images of the slides were captured using a digital microcamera (Electronic Eyepiece Camera Video, Concórdia, Brazil) connected to a biological trinocular microscope (model TNB-41 T-PL, OPTON) and a histological image capture program (Images J). Further details of the methodology used to measure villus height and crypt depth were described by Galli et al. (20).

Segments of jejunum and colon (4 samples per pig, around 5 cm length per segment) were collected randomly immediately after slaughter. Intestinal rupture strength was evaluated using a dynamometer (ITFG6005, Instrutemp, São Paulo, Brazil) that provides the ideal force necessary to break the sample. The results were expressed as kilogram-force per centimeter square (kgf/cm).

#### 2.3.5 Fecal biomarkers

After homogenizing all stool samples collected during the digestibility period, a 5 gram sample was collected and stored in sterile tubes at −20°C until laboratory analysis. Fecal samples were thawed at room temperature for laboratory processing. Briefly, 5 mL of phosphate-buffered saline (PBS) was added to each tube containing a subsample of 100 mg of feces and mixed thoroughly. After that, tubes were centrifuged for 25 min at 400 rpm and after that, it was used vortexed to mix the samples again. A total of 1 mL of each sample was transferred to a microcentrifuge tube and centrifuged for 20 min at 1500 rpm. The supernatant was collected and stored at −20°C until analysis. The detection of porcine calprotectin and intestinal fatty acid-binding proteins was performed using commercial ELISA tests (My Biosource, San Diego, United States), following the manufacturer’s protocol and the Multiskan Sky machine from Thermo Scientific.

### 2.4 Statistical analysis

Analysis of variance was performed using the General Linear Model using Minitab 18 software. The fixed effects of diet type, enzyme addition, and their interactions were considered in the analytical models. Body weight was considered as a co-variable. Fecal moisture was also analyzed by considering the effect of sampling time (days) in the model. All residuals were submitted to the Ryan-Joiner test to assess their normal distribution. Eventual differences among treatments were assessed with the Tukey multiple comparison test and then interpreted at *p* ≤ 0.05 (significant differences) and 0.05 < *p* ≤ 0.10 (the trend for difference).

## 3 Results

### 3.1 General results

The average ambient room temperature was 25.1°C and the average daily relative humidity was 78%. These values suggested that the pigs were housed under thermoneutral conditions. No pigs were removed from the experiment and no health issues were detected during the experimental period.

No interaction between diet type and enzyme addition was found for any of the studied variables (*p* > 0.10). For that reason, these effects were explored separately in this manuscript. An exception was made for the calculated metabolizable energy value because the treatments were formulated with different initial conditions for this response (a reduction of 90 kcal/kg of metabolizable energy was applied in the enzyme treatments).

### 3.2 Feeding response

Feed allotment was determined by considering the body weight of each pig individually at the end of the adaptation period. Consequently, a tendency between complex and simple diets was found in this variable (the feed amount supplied to the animals was 33 g higher for complex diets than for simple diets; *p* = 0.086; Table 2), with no variation among enzyme additions. However, pigs fed simple and complex diets had similar feed intake during the sampling period. No differences among enzyme additions were identified for feeding allotments. The intake adaptation period was 455 g and the coefficient of variation was 12.5%.

**Table 2:** Daily feed allotment, leftovers, and estimated intake in weaned pigs fed diets differing in complexity and enzyme addition^1^.

Item	Diet type		Enzyme addition			RSE^2^	*p-*values^3^		
	Simple	Complex	Control	BM	BM + MCC		Diet	Enzyme	Diet × Enzyme
Initial weight	7.894	7.989	7.971	7.921	7.933	0.290	0.778	0.991	0.999
Feed allotment, g/day	753.6	786.8	779.5	776.9	754.1	50.29	0.086	0.461	0.910
Leftovers, g/day	88.16	78.64	68.68	96.70	84.80	45.75	0.146	0.414	0.856
Intake, g/day	667.6	715.63	721.7	681.7	671.6	64.62	0.572	0.209	0.627

1 Enzyme addition: Without any addition (Control) or addition with β-mannanase (BM; 0.300 g/kg of the diet) or β-mannanase plus a multi-carbohydrase complex containing xylanase, β-glucanase, and arabinofuranosidases (BM + MCC; 0.300 + 0.050 g/kg of the diet).

2 RSE: Residual Standard Error.

3 *p*-value: Probabilities for effects of diet type (Diet; simple and complex), enzyme addition (EA; Control, BM, and BM + MCC), and their interaction (Diet × Enzyme).

Means followed by different uppercase letters differ statistically at the p ≤ 0.05 level, while lowercase letters were used to indicate a trend for 0.05 < p ≤ 0.10.

### 3.3 Digestibility and metabolism

The BM addition plan improved (*p* < 0.01) the energy digestibility coefficient by 2% compared to the control treatment, while an even higher improvement (4%) was found for the BM + MCC treatment in comparison to the control treatment. The protein digestibility coefficient differed between diet types, with a 2% greater value observed for simple than complex diets (Table 3; *p* < 0.05). Regarding enzyme addition, the dry matter and protein digestibility coefficients were similar between BM and BM + MCC, and both coefficients were greater in BM + MCC than in the control treatment (2 and 3% higher, respectively; *p* < 0.05).

**Table 3:** Coefficients (%) of digestibility and metabolizability observed in weaned pigs fed diets differing in complexity and enzyme addition^1^.

Item	Diet type		Enzyme addition			RSE^2^	*p-*values^3^		
	Simple	Complex	Control	BM	BM + MCC		Diet	Enzyme	Diet × Enzyme
Dry matter digestibility	91.09	90.35	89.90^B^	90.28^AB^	91.98^A^	1.861	0.297	0.042	0.946
Protein digestibility	89.45	87.41	87.11^B^	88.28^AB^	89.91^A^	2.327	0.028	0.039	0.831
Energy digestibility	89.75	89.23	87.65^C^	89.79^B^	91.03^A^	1.779	0.469	0.003	0.766
Protein metabolizability	87.91	86.16	85.79^b^	86.98^ab^	88.33^a^	2.183	0.047	0.056	0.905
Energy metabolizability	86.06	85.79	84.94	85.47	87.36	2.739	0.790	0.147	0.799

1 Enzyme addition: Without any addition (Control) or addition with β-mannanase (BM; 0.300 g/kg of the diet) or β-mannanase plus a multi-carbohydrase complex containing xylanase, β-glucanase, and arabinofuranosidases (BM + MCC; 0.300 + 0.050 g/kg of the diet).

2 RSE: Residual standard error.

3 *p*-value: Probabilities for effects of diet type (Diet; simple and complex), enzyme addition (EA; Control, BM, and BM + MCC), and their interaction (Diet × Enzyme).

Means followed by different uppercase letters differ statistically at the *p* ≤ 0.05 level, while lowercase letters were used to indicate a trend for 0.05 < *p* ≤ 0.10.

BM addition improved (*p* < 0.05) the energy metabolizability coefficient by 1% compared to the control treatment, while a higher improvement (3%) was found for the BM + MCC treatment in comparison to the control treatment. The metabolizability coefficient of protein was 2% greater in simple compared to complex diet (*p* < 0.01). When comparing enzyme addition, BM + MCC had a metabolizability coefficient of protein similar to that of BM and tended to be greater than that of the control (3%; *p* = 0.056).

The use of a simple diet resulted in higher fecal nitrogen (*p* < 0.01; Table 4) and lower energy intake and fecal energy (*p* < 0.01; Table 5) compared to a complex diet. Fecal nitrogen and energy intake were lower in BM + MCC than in control (*p* < 0.01). Fecal energy was lower in BM + MCC compared to BM and control treatments (p < 0.01).

**Table 4:** Nitrogen balance observed in weaned pigs fed diets differing in complexity and enzyme addition^1^.

Item	Diet type		Enzyme addition			RSE^2^	*p-*values^3^		
	Simple	Complex	Control	BM	BM + MCC		Diet	Enzyme	Diet × Enzyme
Nitrogen Intake (g/day)	23.22	24.45	24.89	23.48	23.13	2.187	0.133	0.186	0.610
Fecal nitrogen (g/day)	3.209	2.558	3.185^A^	2.928^AB^	2.538^B^	0.434	0.001	0.009	0.522
Urinary nitrogen (g/day)	0.303	0.254	0.328	0.204	0.302	0.206	0.547	0.414	0.299
Absorbed (g/day)	20.49	20.88	21.13	20.39	20.55	1.955	0.597	0.697	0.557
Retained (g/day)	20.22	20.87	20.82	20.87	19.94	1.569	0.297	0.350	0.294
Ratio ret./abs.^4^ (%)	99.56	99.44	98.53	99.88	100.1	0.861	0.731	0.102	0.271

Means followed by different uppercase letters differ statistically at the *p* ≤ 0.05 level, while lowercase letters were used to indicate a trend for 0.05 < *p* ≤ 0.10.

1 Enzyme addition: Without any addition (Control) or addition with β-mannanase (BM; 0.300 g/kg of the diet) or β-mannanase plus a multi-carbohydrase complex containing xylanase, β-glucanase, and arabinofuranosidases (BM + MCC; 0.300 + 0.050 g/kg of the diet).

2 RSE: Residual Standard Error.

3 *p*-value: Probabilities for effects of diet type (Diet; simple and complex), enzyme addition (EA; Control, BM, and BM + MCC), and their interaction (Diet × Enzyme).

4 Ratio ret./abs.: ratio between protein retention and protein absorption.

**Table 5:** Energy balance observed in weaned pigs fed diets differing in complexity and enzyme addition^1^.

Item	Diet type		Enzyme addition			RSE^2^	*p-*values^3^		
	Simple	Complex	Control	BM	BM + MCC		Diet	Enzyme	Diet × Enzyme
Energy Intake (g/day)	2,418	2,571	2646^a^	2436^ab^	2400^b^	232.2	0.083	0.057	0.621
Fecal energy (g/day)	250.6	287.5	302.5^A^	275.4^A^	229.2^B^	38.93	0.021	0.002	0.692
Urinary energy (g/day)	71.05	78.24	92.11	67.35	64.49	41.94	0.666	0.323	0.327
ED^4^ (kcal/kg)	3,250	3,210	3,223	3,212	3,254	65.72	0.134	0.380	0.706
EM^5^ (kcal/kg)	3,129	3,086	3,113	3,087	3,123	90.63	0.232	0.700	0.839
Ratio EM/ED (%)	96.82	96.82	95.99	97.24	97.23	2.294	0.996	0.403	0.663

Means followed by different uppercase letters differ statistically at the *p* ≤ 0.05 level, while lowercase letters were used to indicate a trend for 0.05 < *p* ≤ 0.10.

1 Enzyme addition: Without any addition (Control) or addition with β-mannanase (BM; 0.300 g/kg of the diet) or β-mannanase plus a multi-carbohydrase complex containing xylanase, β-glucanase, and arabinofuranosidases (BM + MCC; 0.300 + 0.050 g/kg of the diet).

2 RSE: Residual Standard Error.

3 *p*-value: Probabilities for effects of diet type (Diet; simple and complex), enzyme addition (EA; Control, BM, and BM + MCC), and their interaction (Diet × Enzyme).

4 ED: digestible energy.

5 EM: metabolizable energy.

**Figure 1:**
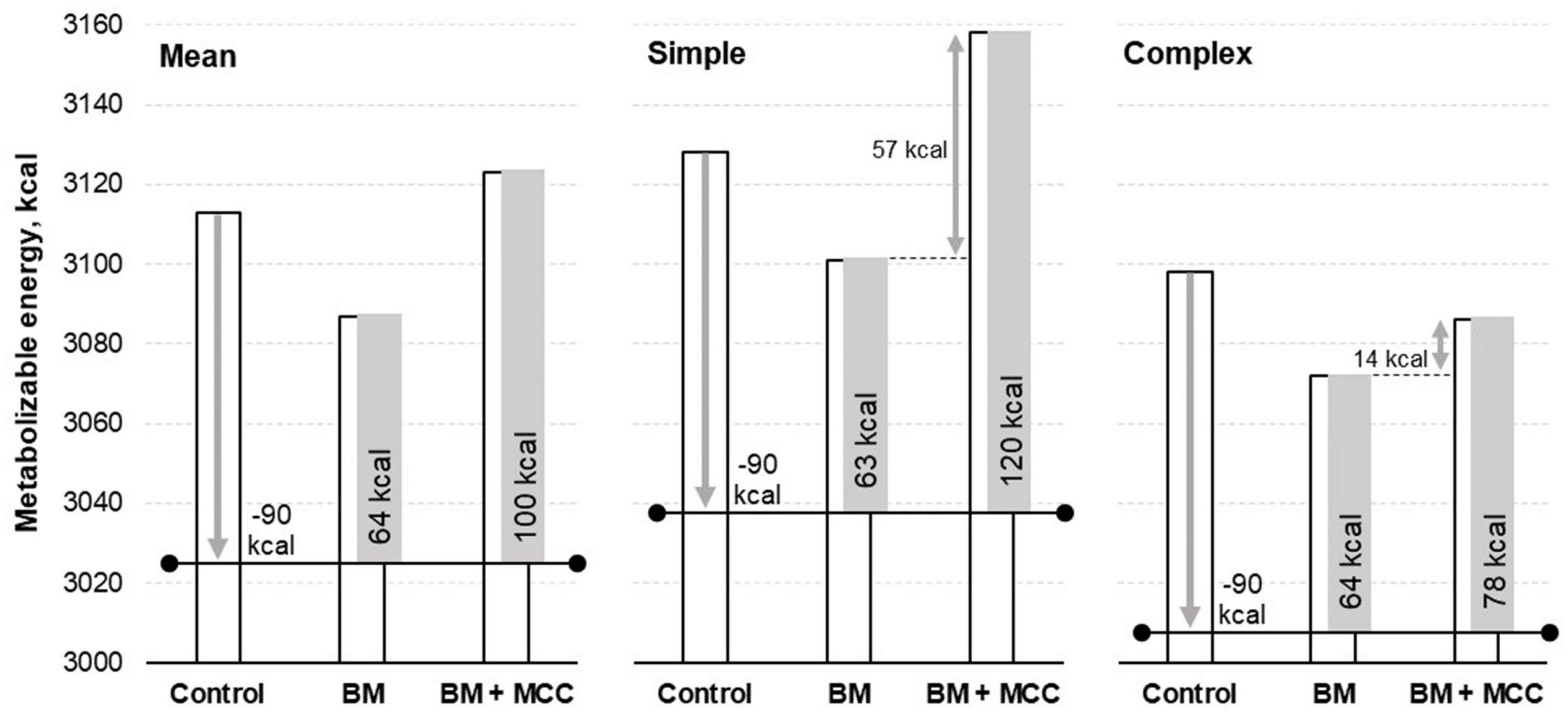
Metabolizable energy observed in weaned pigs fed diets differing in complexity and enzyme addition. Enzyme addition: Without any addition (Control) or addition with β-mannanase (BM; 0.300 g/kg of the diet) or β-mannanase plus a multi-carbohydrase complex containing xylanase, β-glucanase, and arabinofuranosidases (BM+MCC; 0.300 + 0.050 g/kg of the diet).

There was no difference in the enzyme addition for the final digestible and metabolizable energy values. These results need to be interpreted considering that both enzymes’ diets (BM and BM + MCC) were formulated to contain 90 kcal/kg less metabolizable energy than the control diet (energy matrix). Thus, it is possible to infer that these enzymes improved the energy available to the pigs, especially in the simple diet, in which BM could be used with a matrix of 63 kcal/kg of metabolizable energy and BM + MCC combined use resulted in a matrix of 120 kcal/kg of metabolizable energy; however, it is a numerical difference (Figure 1). The ratio between metabolizable and digestible energy was similar among the treatments.

### 3.4 Production of manure, feed retention rate, and fecal moisture content

The feed retention rate was greater in pigs fed the simple diet than in those fed the complex diet (*p* < 0.01). Compared to enzyme addition, BM addition resulted in a lower feed retention rate than the other treatments (*p* = 0.076).

The use of a simple diet resulted in lower fecal production (dry-matter basis) than that of a complex diet (*p* < 0.01; Table 6). Regarding enzyme addition, treatment with BM + MCC resulted in lower fecal production compared to BM and the control (*p* < 0.01), and no difference was found between BM and the control. The production of urine and manure (feces plus urine, both on a dry-matter basis) was similar among the treatments.

**Table 6:** Production of manure (dry matter basis) and feed retention rate observed in weaned pigs fed diets differing in complexity and enzyme addition^1^.

Item	Diet type		Enzyme addition			RSE^2^	*p-*values^3^		
	Simple	Complex	Control	BM	BM + MCC		Diet	Enzyme	Diet × Enzyme
Feed retention rate, min	1,369	1,039	1250^a^	1082^b^	1279^a^	189.0	0.027	0.076	0.231
Feces (g/day)	56.26	65.39	64.66^A^	64.16^A^	53.65^B^	8.918	0.012	0.016	0.675
Urine (g/day)	34.35	34.88	37.96	33.57	32.31	2.118	0.947	0.828	0.579
Manure (g/day)	94.52	100.0	102.4	102.1	87.31	22.29	0.512	0.252	0.551

Means followed by different uppercase letters differ statistically at the *p* ≤ 0.05 level, while lowercase letters were used to indicate a trend for 0.05 < *p* ≤ 0.10.

1 Enzyme addition: Without any addition (Control) or addition with β-mannanase (BM; 0.300 g/kg of the diet) or β-mannanase plus a multi-carbohydrase complex containing xylanase, β-glucanase, and arabinofuranosidases (BM + MCC; 0.300 + 0.050 g/kg of the diet).

2 RSE: Residual Standard Error.

3 *p*-value: Probabilities for effects of diet type (Diet; simple and complex), enzyme addition (EA; Control, BM, and BM + MCC), and their interaction (Diet × Enzyme).

**Figure 2:**
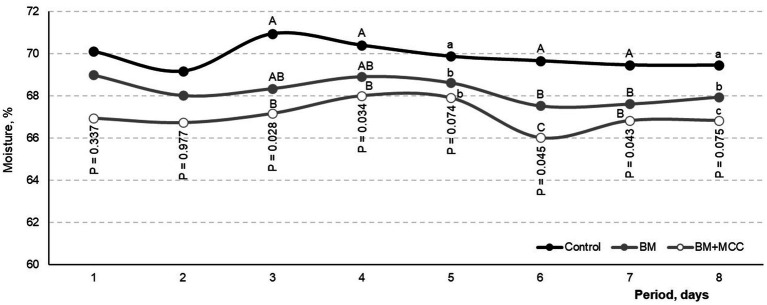
Moisture content in feces observed in weaned pigs fed diets differing in complexity and enzyme addition. Mean observed for control treatment was 69.88% (No addition), which differed from BM with 68.25% (β-mannanase). Both previous treatments differed from BM + MCC with 67.05% (BM+MCC). Day 1 corresponds to the first day of the collection period. Pigs were fed experimental diets during the adaptation period (before day 1). Enzyme addition: Without any addition (Control) or addition with β-mannanase (BM; 0.300 g/kg of the diet) or β-mannanase plus a multi-carbohydrase complex containing β-glucanase, xylanase, β-glucanase, and arabinofuranosidases (BM+MCC; 0.300 + 0.050 g/kg of the diet).

No severe diarrhea (> 80% moisture) was observed during the trial. However, the moisture content of the feces in the control treatment (mean value: 69.88%) was greater (*p* < 0.05) than that in the BM (68.25%) and BM + MCC (67.05%; Figure 2). The sampling periods differed in terms of fecal moisture (*p* < 0.01); however, no differences were found between diet types, and no interactions were found for this variable.

### 3.5 Acute phase proteins and fecal biomarkers

Serum concentrations of haptoglobin were similar between BM + MCC and BM, while both treatments had lower haptoglobin concentrations than control pigs (Table 7). Otherwise, C-reactive protein concentrations were not affected by treatments. Transferrin (average for all the treatments 35 mg/dL) was removed from the table because the measure did not work.

**Table 7:** Acute phase proteins and fecal biomarkers observed in weaned pigs fed diets differing in complexity and enzyme addition^1^.

Item	Diet type		Enzyme addition			RSE^2^	*p-*values^3^		
	Simple	Complex	Control	BM	BM + MCC		Diet	Enzyme	Diet × Enzyme
C-reactive protein, mg/dL	1.120	1.040	1.080	1.080	1.080	0.190	0.268	0.998	0.140
Haptoglobin, mg/Dl	10.20	10.20	11.00^a^	8.700^b^	9.900^b^	1.400	0.998	0.087	0.357
Calprotectin (ng/mL)	41.31	38.68	53.36^A^	38.06^B^	28.56^B^	19.17	0.558	<0.001	0.775
Intestinal fatty acid-binding proteins (pg/mL)	198.4	188.6	211.7	191.7	177.2	53.99	0.533	0.207	0.634

Means followed by different uppercase letters differ statistically at the *p* ≤ 0.05 level, while lowercase letters were used to indicate a trend for 0.05 < *p* ≤ 0.10.

1 Enzyme addition: Without any addition (Control) or addition with β-mannanase (BM; 0.300 g/kg of the diet) or β-mannanase plus a multi-carbohydrase complex containing xylanase, β-glucanase, and arabinofuranosidases (BM + MCC; 0.300 + 0.050 g/kg of the diet).

2 RSE: Residual Standard Error.

3 *p*-value: Probabilities for effects of diet type (Diet; simple and complex), enzyme addition (EA; Control, BM, and BM + MCC), and their interaction (Diet × Enzyme).

The intestinal fatty acid-binding proteins were not affected by diet type or enzyme addition. Pigs fed simple or complex diets also showed comparable fecal calprotectin concentrations. However, both enzyme additions greatly reduced the calprotectin concentration compared to the control treatment (*p* < 0.001). The fecal calprotectin concentration observed in pigs with BM addition was 29% lower than that of the control, while the use of the BM + MCC combination produced an even greater reduction, reaching 46% in relation to the control.

### 3.6 Intestinal rupture resistance and morphology

The gut resistance to rupture was not affected the by type of diet and enzyme addition (Table 8). Nevertheless, the simple diets resulted in lower villus height (*p* < 0.001), villus area (*p* < 0.05), and villus:crypt ratio (*p* < 0.001) compared to complex diet. Regarding enzyme addition, BM + MCC resulted in greater villus height (*p* < 0.001) and villus area (*p* = 0.075), compared to BM and control, and no differences were found between BM and control for both variables.

**Table 8:** Gut resistance and intestinal morphology observed in weaned pigs fed diets differing in complexity and enzyme addition^1^.

Item	Diet type		Enzyme addition			RSE^2^	*p-*values^3^		
	Simple	Complex	Control	BM	BM + MCC		Diet	Enzyme	Diet × Enzyme
Jejunum, kgf	2.133	2.021	2.072	2.150	2.012	0.494	0.590	0.357	0.149
Colon, kgf	1.772	1.860	1.772	1.780	1.901	0.416	0.418	0.358	0.292
Villi height, μm	388.5	413.9	397.2^B^	384.1^B^	422.5^A^	38.23	<0.001	<0.001	0.152
Villi width, μm	123.9	126.7	125.0	127.0	123.9	16.60	0.212	0.518	0.224
Villi area, μm^2^	48,562	52,647	50114^b^	49038^b^	52663^a^	11,709	0.003	0.075	0.674
Crypt depth, μm	328.9	323.3	321.4	326.1	332.1	92.79	0.548	0.498	0.165
Ratio villi height: crypt depth	1.320	1.410	1.354	1.344	1.399	0.506	<0.001	0.501	0.150

Means followed by different uppercase letters differ statistically at the *p* ≤ 0.05 level, while lowercase letters were used to indicate a trend for 0.05 < *p* ≤ 0.10.

1 Enzyme addition: Without any addition (Control) or addition with β-mannanase (BM; 0.300 g/kg of the diet) or β-mannanase plus a multi-carbohydrase complex containing xylanase, β-glucanase, and arabinofuranosidases (BM + MCC; 0.300 + 0.050 g/kg of the diet).

2 RSE: Residual Standard Error.

3 *p*-value: Probabilities for effects of diet type (Diet; simple and complex), enzyme addition (EA; Control, BM, and BM + MCC), and their interaction (Diet × Enzyme).

## 4 Discussion

The current study was performed to evaluate whether adding β-mannanase alone or in combination with arabinofuranosidases, β-glucanase, and xylanases to simple and complex diets could improve diet digestibility, nutrient metabolism on post-weaned pigs’ intestinal health. Although both enzymes are used with an energy matrix in feed formulation, we also hypothesized that these additives could have additive effects, as they perform different functions in the animal, such as saving energy that would otherwise be spent on the immune response (β-mannanases) or releasing nutritional components for absorption (arabinofuranosidases, β-glucanase, and xylanases). Hence, we hypothesized that these enzymes could produce greater effects on the intestinal health of post-weaned pigs. We also hypothesized that a simple diet could replace a complex diet, without compromising energy, nutrient metabolism, and intestinal health.

The addition of BM and BM + MCC improved the dry matter, protein, and energy digestibility coefficients. The effects were greater when BM + MCC was added, suggesting an additive effect between the enzymes. The increase in the nutrient digestibility coefficient may occur because β-mannanase reduces the viscosity of digesta promoted by β-mannans, allowing better contact of the digesta with the gut (21). In agreement, Jeon et al. (22) reported that dietary addition of 0.05% β-mannanase resulted in greater standardized ileal digestibility of amino acids, total tract apparent digestibility, and crude protein for growing pigs. Lu et al. (4) observed that the addition of xylanase to post-weaned pigs improved dry matter and crude energy digestibility. These findings may be associated with xylanase releasing nutrients trapped inside the cell wall of cereal grains, primarily arabinoxylans, which are found in corn’s aleurone and the starchy endosperm. Furthermore, increased digestibility is usually associated with increased energy digestibility (23). Digestible energy, on the other hand, is not a nutrient *per se* but rather the sum of improved digestibility of starch, fat, protein, and fiber (9). Duarte et al. (11) found that adding 45,000 U/kg xylanase to the diet of post-weaned pigs reduced digesta viscosity in the jejunum. As a result, by reducing viscosity, enzymes’ access to substrates can be increased.

Moreover, enzyme addition increased the protein and energy metabolizability coefficients. On average, BM provides 64 kcal/kg of metabolizable energy, while BM + MCC provides 100 kcal/kg. BM addition showed a very similar metabolizable energy matrix in simple (63 kcal/kg) or complex diets (64 kcal/kg). However, the matrix of metabolizable energy calculated for combined enzymes was greater in the simple diet (120 kcal/kg) than in the complex diet (78 kcal/kg), which is probably related to the greater amount of substrate for the enzyme blend to act on. These results support the hypothesis that these enzymes can have complementary effects, despite having energy matrices.

Complex diets are defined by the higher inclusion of more palatable and digestible ingredients, such as whey, plasma, viscera meal, and soy isolate (24). While simple diets include more soybean meal is the main protein source in swine diets because of its high protein and amino acid content, consistent quality, and availability in the market. However, soybeans contain anti-nutritional factors such as protease inhibitors and mannans, which interfere with the digestion and absorption of nutrients. In this trial, the simple diet showed greater protein digestibility and metabolizability coefficients than the complex diet. It is important to highlight that this was probably a consequence of the ingredient composition. Both diets were formulated considering metabolizable energy and digestible amino acid values, and the crude contents were similar between simple and complex diets. The simple diet had a higher inclusion of soybean oil compared to the complex diet. It is known that the inclusion of lipids improves the digestibility of nutrients because it increases the secretion of cholecystokinin in the intestine, pancreatic juice, and enzymatic activity. This could explain our finding of greater protein digestibility in the simple diet.

Although this is a methodological limitation, no other option was available once different ingredients were used to generate simple and complex diets. As a direct comparison between simple and complex diets is not a direct objective of the trial, these results need to be interpreted with caution. Apart from the formulation procedure, this difference may be also related to the faster passage rate of the complex diet as the feed retention rate increases (25, 26). Ratanpaul et al. (27) reported that feed intake decreases as the feed retention rate increases. This is consistent with the findings of the present study. Furthermore, a higher retention rate allows endogenous enzymes to act for a longer period in the digesta (27). Other possible mechanisms that could explain our findings include gastric distension and feedback effects on the hypothalamus, which reduce the gastric emptying rate (28).

Zhao et al. (29) reported that soluble dietary fiber has a high ability to bind water and thereby increase viscosity, thus increasing fecal mass and moisture content. One possible explanation could be that the addition of BM and BM + MCC reduced the amount of non-starch polysaccharides in the digesta, resulting in less fiber available to bind with water, thus explaining the lower moisture in the feces of the pigs that were fed the exogenous enzymes. Even after adjusting fecal production to a dry matter basis, the addition of BM + MCC reduced fecal production by 17%. This is possibly due to the feed retention rate and better digestibility coefficients, as previously mentioned, and is a positive result when considering the potential mitigation of the environmental impact of pig production.

A trend toward lower haptoglobin concentrations was found when BM and BM + MCC treatments were compared to the control. Haptoglobin is a major acute-phase protein in pigs and an increase in its plasma concentration is an indicator of systemic inflammation (30). Huntley et al. (31) observed a decrease in serum haptoglobin concentration after administering β-mannanase to post-weaned pigs, indicating that β-mannanase reduced the immune response caused by β-mannans. Furthermore, McAfee et al. (32) reported that it is related to the control of inflammatory responses.

Biomarkers are useful for identifying various gastrointestinal conditions, particularly non-invasive biomarkers that can avoid animal euthanasia. Calprotectin is a cytosolic protein composed of S100A8 and S100A9 (33). This protein binds to calcium and zinc and is found in neutrophil granulocytes (34). Calprotectin is a useful biomarker because when neutrophils congregate at sites of inflammation, they produce more calprotectin (35). Calprotectin is already used in human health, but it is thought to be a promising biomarker of pig intestinal inflammation (36). The literature on the effects of additives on pig calprotectin concentrations still very limited. Xiao et al. (37) reduced calprotectin concentrations by adding chitosan to post-weaning pig diets. To the best of our knowledge, the present study is the first to test calprotectin in post-weaned pigs in the context of enzyme addition. The addition of both enzymes greatly reduced calprotectin concentrations. Thus, it is possible to infer that enzyme addition reduced inflammation caused by dietary anti-nutritional factors such as β-mannans, arabinoxylans, xylose, trypsin, and others.

Gut morphology is also used as an indicator of gut health (30). Increased villus height is usually interpreted as a positive trait of gut morphology and is also used as an indicator of gut health (30). In the current study, both responses (villus height and villus area) improved in pigs fed a complex diet. Some physical properties of a simple feed (e.g., more abrasive) may be important to understand this difference. The enzymatic addition of BM + MCC also improved the same responses, which correlated with an improved nutrient digestibility coefficient.

Simple diets are a suitable alternative for the nursery phase if well-formulated. The use of simple feeds is a way to further explore the potential of BM + MCC enzyme addition since its effects were more pronounced in simple diets for some responses, such as metabolizable energy. The effects of the addition of both enzymes can be greater when working with the enzyme combination since both have an energy matrix. It was observed that both enzymes collaborated to the lower inflammation status assessed in this study through the fecal calprotectin concentrations. To the best of our knowledge, no studies are available focusing on dietary complexity and correlating this promising biomarker for gut health with animal performance. Exploring growth performance was not feasible in our trial due to the limitation imposed by housing conditions (metabolic crates), but it would be highly significant for future research. Additionally, our trial focused on pigs that were weaned at 28 days. It is advisable to interpret these results cautiously when considering scenarios with younger weaning ages.

Feeding strategies using simple diets reduce feed costs in the swine industry because of the reduced inclusion of expensive special ingredients (i.e., spray-dried spray, whey powder, and protein isolates). Furthermore, the addition of exogenous enzymes reduced the amount of soybean oil in the feed formulas. This reduction could potentially mitigate environmental impacts, as soybean oil is generally associated with a high potential for global warming. The environmental and economic implications of simplifying the diets are highly dependent on the scenario in which the modification is performed; however, this feeding strategy could be explored by swine nutritionists as a viable alternative for nursery pigs.

## 5 Conclusion

The simple diet had greater protein digestibility and metabolizability coefficients than a complex diet. A complex diet had greater villus height and villus area than a simple diet. Both enzyme additions increased energy digestibility, with BM addition releasing 64 kcal and BM + MCC releasing 100 kcal of metabolizable energy per kg of feed. The addition of BM and BM + MCC also reduced the calprotectin and haptoglobin concentrations and improved the absorption area.

## Data availability statement

The raw data supporting the conclusions of this article will be made available by the authors, without undue reservation.

## Ethics statement

The animal studies were approved by The experimental protocol was approved by the institutional ethics committee on the use of animals (CEUA/UFRGS – Protocol 39,604). The studies were conducted in accordance with the local legislation and institutional requirements. Written informed consent was obtained from the owners for the participation of their animals in this study.

## Author contributions

GG: Conceptualization, Data curation, Formal analysis, Investigation, Methodology, Project administration, Validation, Visualization, Writing – original draft, Writing – review & editing. IA: Conceptualization, Formal analysis, Funding acquisition, Investigation, Project administration, Software, Supervision, Validation, Visualization, Writing – review & editing. CC: Data curation, Formal analysis, Methodology, Writing – review & editing. TS: Data curation, Formal analysis, Methodology, Writing – review & editing. MM: Data curation, Formal analysis, Methodology, Writing – review & editing. RM: Formal analysis, Methodology, Writing – review & editing. VH: Formal analysis, Methodology, Writing – review & editing. MK: Funding acquisition, Project administration, Resources, Supervision, Visualization, Writing – review & editing.
